# 302 A Classification System and Surgical Algorithm for Pediatric Postburn Ear Deformities

**DOI:** 10.1093/jbcr/irad045.277

**Published:** 2023-08-29

**Authors:** Martin Buta, Branko Bojovic, Matthew DePamphilis, Daniel N N Driscoll, Richard Ehrlichman

**Affiliations:** Massachusetts General Hospital, Boston, Massachusetts; Massachusetts General Hospital, Boston, Massachusetts; Boston University School of Medicine, Boston, Massachusetts; Massachusetts General Hospital, Boston, Massachusetts; Massachusetts General Hospital, Boston, Massachusetts

## Abstract

**Introduction:**

The outward projection of the external ear makes this anatomically complex appendage susceptible to trauma. Notably, up to 40-60% of facial burns involve the ear, frequently impacting function and aesthetics. Significant postburn sensory deficits and changes to the ear’s intricate architecture can markedly impair physical health, psychosocial functioning, and quality of life. Scar tissue and contracture, traumatized cartilage, and compromised blood supply and surrounding soft tissues all influence the reconstructive plan. The authors describe a new classification system and surgical algorithm for pediatric postburn ear deformities informed by both their extensive operative experience on the traumatized ear and a retrospective study of different reconstructive techniques for more severe burn injuries to the pediatric ear.

**Methods:**

To develop the classification system and surgical algorithm, three senior surgeons with over 85 years of combined experience reconstructing ears carried out an iterative joint review of their relevant clinical cases and insights, including their personal approach to assessing postburn ear trauma and developing an operative plan. Particular attention was focused on how the type and extent of soft tissue injury affects available reconstructive options. To inform classification of the most complex ear burn cases, a retrospective review was conducted on patients aged 0 to 21 years who underwent cartilage framework reconstruction between January 2004 to January 2021 at a specialized pediatric burn center. Medical records were screened and 54 patients (61 ears) who met study criteria were identified and patient demographics, procedural characteristics, and outcomes were analyzed.

**Results:**

For postburn ear deformities (Table 1) involving total or near-total preservation of the ear’s anatomical structural elements (Type I and Type II deformities, respectively), scar release, local tissue rearrangement, and/or skin grafting are typically sufficient for optimal reconstruction. For cartilage defects involving less than or more than two-thirds of the helix (Type III and Type IV deformities, respectively), a partial to complete ear scaffold can be successfully reconstructed using a composite graft, conchal transposition flap, costal cartilage graft, or porous polyethylene implant.

**Conclusions:**

The proposed classification system and surgical algorithm (not shown) for postburn ear deformities facilitate a tailored approach that addresses reconstructive course duration, anticipated aesthetic results, complication potential, and patient goals and expectations.

**Applicability of Research to Practice:**

The authors provide a new classification system to pediatric postburn ear deformities that, along with a proposed surgical algorithm, can help guide planning for reconstruction.

